# Supplementary material for: Deep learning image segmentation reveals patterns of UV reflectance evolution in passerine birds
Source: Nat Commun. 2022 Aug 29;13:5068. doi: 10.1038/s41467-022-32586-5 (PMC9424304; doi:10.1038/s41467-022-32586-5)
Supplement: Supplementary file 1 — Supplementary Information [file 41467_2022_32586_MOESM1_ESM.pdf]

SUPPLEMENTARY INFORMATION

for

Deep learning image segmentation reveals patterns of UV reflectance evolution in passerine birds

Authors: Yichen He*, Zoë K. Varley, Lara O. Nouri, Christopher J. A. Moody, Michael D. Jardine, Steve Maddock, Gavin H. Thomas*, Christopher R. Cooney*

*Corresponding authors. Email: yhe20@sheffield.ac.uk, gavin.thomas@sheffield.ac.uk, c.cooney@sheffield.ac.uk

Supplementary Note 1. Methods and Results from Additional Model Testing

(i) Effects of input resolution on the performance

DeepLabv2 has been shown to perform better when using the original input resolution compared to resized resolutions[1], in particular downscaled images result in lower accuracy for pose estimation and classification[2]. We compared resolutions that were 8, 10 and 16 times lower than the input images (i.e. 618 x 410, 494 x 328 pixels and 309 x 205 pixels) to test whether performance degrades at lower resolutions.

There was a significant effect of input image resolution on intersection over union (IOU) (ANOVA: F = 1361.0; *d.f.* = 2, 15279; P < 0.01), precision (ANOVA: F = 1069.2; *d.f.* = 2, 15279; P < 0.01) and recall (ANOVA: F = 456.3; *d.f.* = 2, 15279; P < 0.01) (Supplementary Fig. 1). The IOU and recall of 618 x 410 pixels and 494 x 328 pixels were not significantly different from each other, while the rest of the accuracies (IOU, precision and recall) were positively related to the input resolution (Supplementary Fig. 1). The image resolution of 618 x 410 pixels had the best overall performance, while the 309 x 205 pixels had the worst performance.

(ii) Effects of input channels on the performance

Previous studies have included non-visible light (e.g. UV and IR) information as the input in deep learning tasks, sometimes leading to better performance when compared to using only RGB channels[3, 4, 5]. Our dataset includes two sets of images, one filtered to include only human visible (RGB) wavelengths and one to include only UV wavelengths, because bird plumage frequently includes UV reflecting regions. All images were taken against a black background made of theatre blackout curtains with very low reflectance of the UV light. The specimens should therefore reflect more UV light than the background. To test whether the inclusion of UV improved network performance, models were trained with (i) images using RGB channels only, (ii) images using UV channels only and (iii) images using RGB plus UV channels.

There was a significant effect of input channels on IOU (ANOVA: F = 395.6; *d.f.* = 2, 15279; P < 0.01), precision (ANOVA: F = 184.9; *d.f.* = 2, 15279; P < 0.01) and recall (ANOVA: F = 236.6; *d.f.* = 2,15279; P < 0.01) (Supplementary Fig. 2). RGB was consistently better than UV and RGB+UV, although effects tended to be small (evaluation results of UV and RGB+UV were <2% worse than RGB) (Supplementary Fig. 2).

(iii) Effects of image augmentation on the performance

Image augmentation is a common technique that increases the size of the training set by creating new labelled training images from manipulating the existing images and their labels, and has been shown to improve the model performance of DeepLabv2[1]. We created an augmented training set from the original training set in which images and their segmentations were randomly rotated (-15° to -1°, 1° to 15°), translated in both x and y axes (100 to 500 pixels), scaled (0.1 to 1.1). We used the augmented dataset to train the model with evaluation performed on the original validation set.

IOU was marginally, albeit significantly, higher ($t_{[10186]}$ = 5.90, P < 0.05) for the original dataset (mean = 93.1, standard deviation [SD] = 3.24) than for the augmented dataset

(mean = 92.7, SD = 3.35) (Supplementary Fig. 3). We also found a similar marginal yet significant difference in the precision ($t_{[10186]}$ = 6.63, P < 0.05) and again the original dataset (mean = 96.3, SD = 2.38) outperformed the augmented dataset (mean = 96.0, SD = 2.56) (Supplementary Fig. 3). However, there was no significant difference in the recall ($t_{[10186]}$ = 1.81, P = 0.07) (Supplementary Fig. 3). Our original training sets consists of highly standardised sets of images and we suspect that these minor reductions in performance associated with models trained on augmented datasets are caused by the introduction of non-standard (i.e. rotated, translated and scaled) images into the training set.

(iv) Effects of subsetting models on the performance

In our core pipeline, we used one deep learning model on images from all views (all-views model), but image variations of different views may introduce difficulties for the network to learn. We therefore tested the impact of training and validated separate models for each of the three image views (back, belly and side). This reduces the input data for each model run to 1698 images (compared to 5094 images).

We found that subsetting models by image view (i.e. back, belly, side) was significantly worse than using the all-views model, except for recall on the side view ($t_{[3394]}$ = 1.43, P = 0.15) (Supplementary Fig. 4). The back view had the largest IOU difference (the all-views model has 0.7 higher IOU than individual models) and recall difference (recall of the all-views model is 0.5 higher), while the side view had the largest precision difference (precision of the all-views model is 0.4 higher) (Supplementary Fig. 4).

(v) Quality of the training data

In our case, specimen images were taken in a highly consistent manner by controlling the placement of the specimen, light environment and background[6]. However, not all image datasets are likely to be so consistent due to practical limits (e.g. inadequate lighting). We tested whether greater variability in data quality could limit performance by generating artificial lower quality datasets. To do this, we applied a series of image manipulations in which (i) images were rotated (angles between -45° to 45°), translated (-500 to 500 pixels on x and y axes) and scaled (scale ratio from 0.8 to 1.2), (ii) 50% of images were randomly horizontally flipped, (iii) images were given new contrast and brightness (α from 0.5 to 2 and β from -50 to 50) using brightness and contrast adjustment functions in OpenCV[7], and (iv) a combination of manipulations from (i), (ii) and (iii). We applied these operations to both training images and validation images (in contrast to image augmentation outlined above where we did not manipulate the validation set).

Low-quality datasets had a significant negative effect on the IOU (ANOVA: F = 205.3; *d.f.* = 4.0, 25465; P < 0.01), precision (ANOVA: F = 132.8; *d.f.* = 4.0, 25465; P < 0.01) and recall (ANOVA: F = 88.0; *d.f.* = 4.0, 25465; P < 0.01) (Supplementary Fig. 5). The original dataset produced more accurate results than low-quality datasets (Supplementary Fig. 5a). The 4th dataset (the combination of translation, rotation, scale, horizontal flip and manipulations of brightness and contrast) had the worst performance and examples of its predictions are shown in Supplementary Fig. 9). The 4th dataset was 1.9%, 1.2% and 0.9% worse than the original dataset on IOU, precision and recall (Supplementary Fig. 5b).

(vi) Training dataset size

We manually labelled 5094 images for this study. However, the number of labelled images may be limited by time and resources for other projects and studies. Here, we investigated the impact on deep learning accuracy using smaller training sets. Previous studies suggest that larger training set sizes may improve the performances of deep learning models[8, 9]. We used a subset of 1018 images (20% of the dataset) as the only validation set for every result in this section. The training set (4076 images) was randomly sampled five times for one proportion selected from 15 proportions (1%, every 5% from 5% to 50% and every 10% from 50% to 90%).

We found that model performance was positively related to the training set size following an approximately logarithmic pattern (Supplementary Fig. 6). At least 10% of the dataset was required to attain IOU higher than 90%, at least 5% of the dataset to get precision and recall higher than 90%, and 15% of the dataset for precision and recall higher than 95%. With 100% of the dataset used for training, the model achieved 93.3% for IOU, 96.3% for precision and 96.8% for recall (Supplementary Fig. 6).

(vii) Effects of the plumage colour and background contrast on performance

We explored how the contrast between plumage and non-plumage areas within images affected model performance. We assumed that plumages with high colour contrast to the surrounding non-plumage area would be easier to accurately segment in DeepLabv3+ and classic methods (thresholding, region growing, Chan-Vese, Graph Cut) than low contrast plumage areas. This is because humans and many classic segmentation methods usually segment targets well in images with high contrast between the foreground (e.g. plumage area) and the background (e.g. non-plumage area).

To test this, we measured the contrast by first calculating the per-pixel absolute Laplacian derivatives. A large Laplace derivative of a pixel indicates that the pixel is likely to be around the edges of an object or image feature (i.e. high contrast around this pixel), and has been widely used for image edge detection[10, 11]. We used the mean Laplacian derivative of the pixels around the plumage area borders to represent the plumage area contrast. Pixels are selected from the difference between two segmentations based on two morphological transformations, erosion and dilation[12]. Erosion can be seen as zooming in a segmentation and dilation can be seen as zooming out. The 'zooming' effect is positively affected by the size of the transformation kernel (strength) and iterations (length). We used a kernel size of five for one iteration to create eroded and dilated segmentations on OpenCV. A large contrast value means that the plumage area is very different from its surrounding non-plumage area. We then calculated the Pearson's correlation coefficient (r) between accuracy metrics (IOU, precision and recall) and contrast values to evaluate the effect of contrast on performance.

For DeepLabv3+, we found that IOU, precision and recall values were generally high across all levels of contrast but marginally declined with increasing contrast between plumage colouration and the background (Supplementary Fig. 7). This slight decline in model performance with increasing contrast is opposite to the expectation that high-contrast specimens should be easier to segment than low-contrast specimens but can potentially be explained by lower sample sizes at higher contrasts resulting in more limited

opportunities for our model to learn to accurately segment high-contrast birds. For classic methods (thresholding, region growing, Chan-Vese, Graph Cut), results were more mixed but generally conform to expectations that segmentation performance using threshold-based methods is typically poorer for low-contrast specimens and generally (but not always) improves with increasing contrast (Supplementary Fig. 7).

On the basis of these results, we suggest that Deep Learning approaches to (specimen) image segmentation are generally robust to levels of contrast between specimens and the background, provided a consistent background colour is used and the model is sufficiently well trained on images of varying contrast.

Supplementary Figures

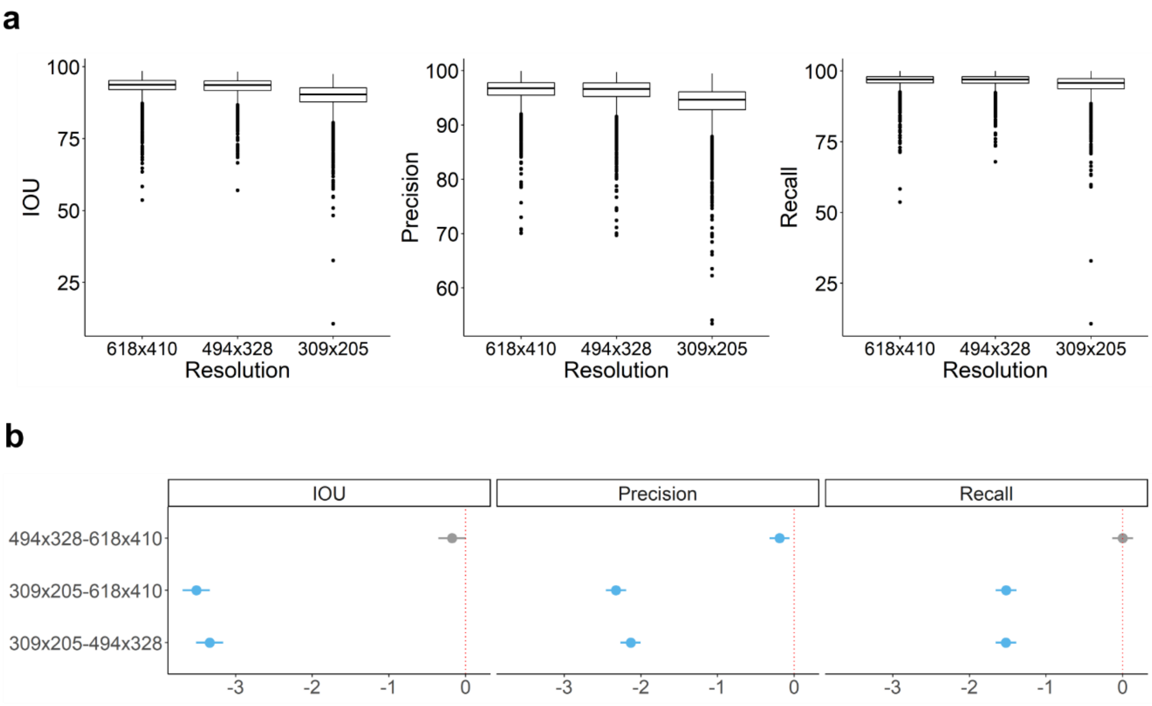

Supplementary Fig. 1. The model performances (IOU, precision and recall) using different input resolutions (618 x 410, 494 x 328 pixels and 309 x 205 pixels). (a) Boxplots of IOU, precision and recall of predictions (*n*=5094). In box plots, a box indicates the median and first and third quartile, whiskers indicate range of data and points indicate outliers. (b) Plots of Tukey's test (95% family-wise confidence level) on whether metric (IOU, precision and recall) differences among tested resolutions (*n*=5094) are significantly different (blue: significance; grey: no significance) from 0 (red dotted lines). In Tukey test plots, a point indicates the mean and whiskers indicate the 95% confidence intervals. Source data are provided as a Source Data file.

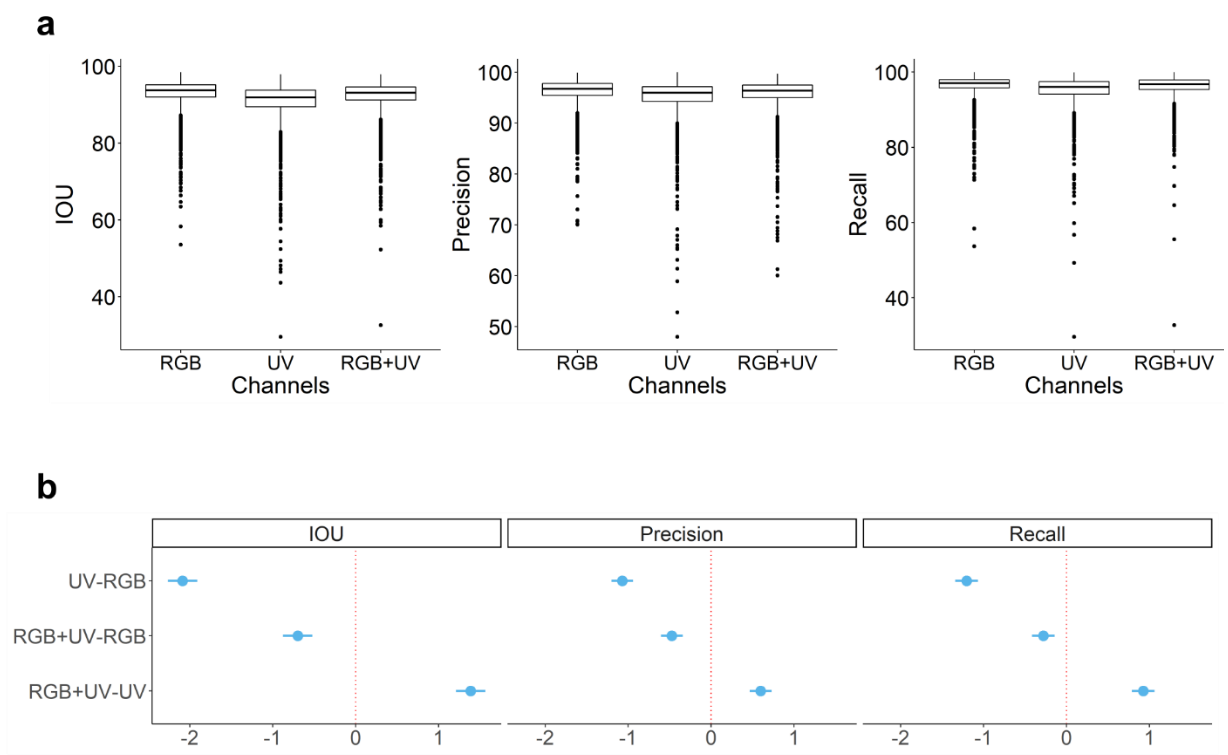

Supplementary Fig. 2. The model performances (IOU, precision and recall) on tested input channels (RGB, UV and RGB+UV). (a) Boxplots of IOU, precision and recall of predictions (*n*=5094). In box plots, a box indicates the median and first and third quartile, whiskers indicate range of data and points indicate outliers. (b) Plots of Tukey's test (95% family-wise confidence level) on whether metric (IOU, precision and recall) differences (*n*=5094) among tested channels (*n*=5094) are significantly different (blue: significance; grey: no significance) from 0 (red dotted lines). In Tukey test plots, a point indicates the mean and whiskers indicate the 95% confidence intervals. Source data are provided as a Source Data file.

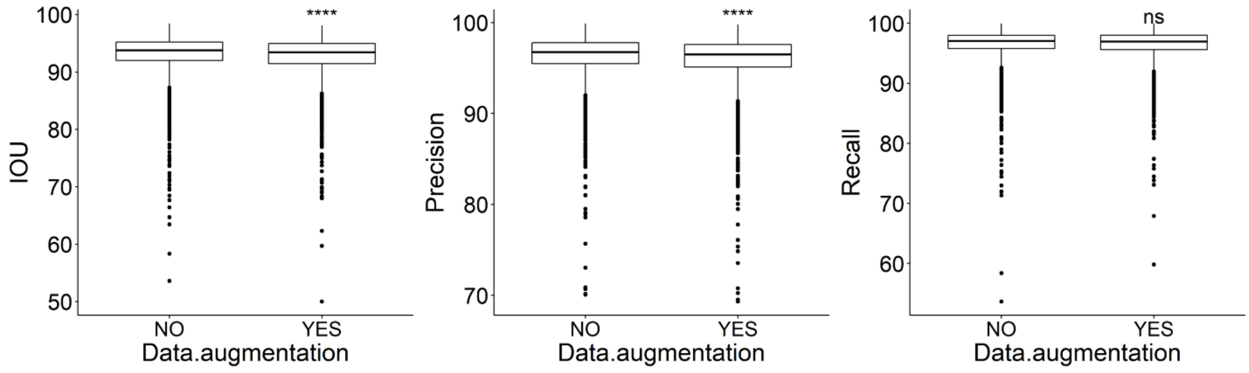

Supplementary Fig. 3. Boxplots of the performances (IOU, precision and recall) of predictions (*n*=5094) between using the original training sets and image augmented training sets. Two-sided t-test is used in all cases. IOU: $t_{(10186)} = 5.90$, $P = 3.80 \times 10^{-9}$; Precision: $t_{(10186)} = 6.63$, $P = 3.45 \times 10^{-11}$; Recall: $t_{(10186)} = 1.81$, $P = 0.07$. Significant symbols are t-test results (ns: $p > 0.05$; *: $p <= 0.05$; **: $p <= 0.01$; ***: $p <= 0.001$; ****: $p <= 0.0001$). In box plots, a box indicates the median and first and third quartile, whiskers indicate range of data and points indicate outliers. Source data are provided as a Source Data file.

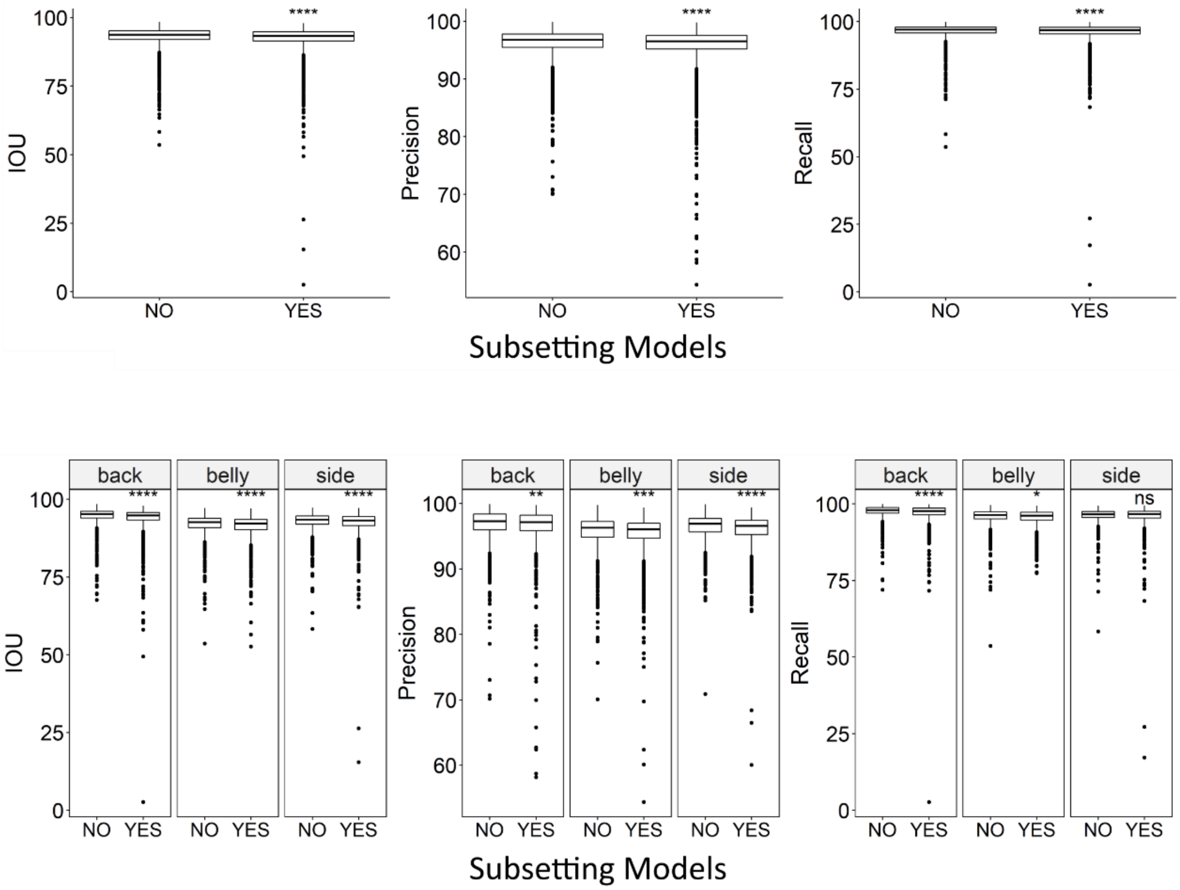

Supplementary Fig. 4. Boxplots of the performances (IOU, precision and recall) of predictions (*n*=5094) between using one model and three separate models. Two-sided t-test is used in all cases. Overall: IOU ($t_{[10186]}$ = 7.98, P = 1.65 x 10^{-15}), Precision ($t_{[10186]}$ = 6.75, P = 1.54 x 10^{-11}) and Recall ($t_{[10186]}$ = 5.19, P = 2.19 x 10^{-7}). Back view: IOU ($t_{[3394]}$ = 5.80, P = 7.28 x 10^{-9}), Precision ($t_{[3394]}$ = 2.89, P = 0.0039) and Recall ($t_{[3394]}$ = 5.53, P = 3.46 x 10^{-8}). Belly view: IOU ($t_{[3394]}$ = 4.22, P = 2.55 x 10^{-5}), Precision ($t_{[3394]}$ = 3.68, P = 0.00023) and Recall ($t_{[3394]}$ = 2.44, P = 0.015). Side view: IOU ($t_{[3394]}$ = 4.36, P = 1.35 x 10^{-5}), Precision ($t_{[3394]}$ = 5.69, P = 1.34 x 10^{-8}) and Recall ($t_{[3394]}$ = 1.43, P = 0.15). Significant symbols are t-test results (ns: $p > 0.05$; *: $p <= 0.05$; **: $p <= 0.01$; ***: $p <= 0.001$; ****: $p <= 0.0001$). In box plots, a box indicates the median and first and third quartile, whiskers indicate range of data and points indicate outliers. Source data are provided as a Source Data file.

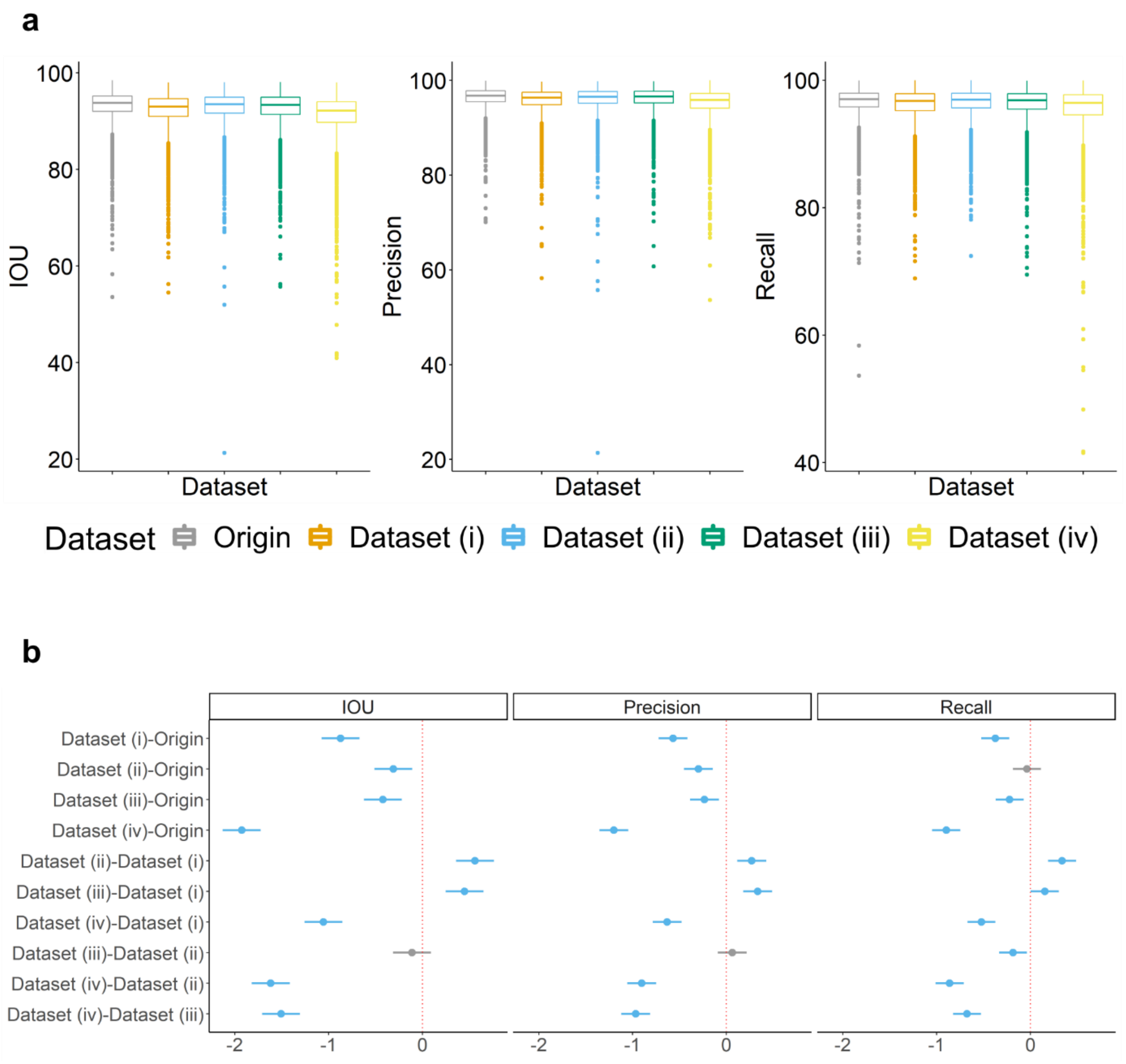

Supplementary Fig. 5. The model performances (IOU, precision and recall) using the original and low-quality datasets. (a) Boxplots of IOU, precision and recall of predictions (*n*=5094). Dataset (i) rotated (angles between -45° to 45°), translated (-500 to 500 pixels on x and y axes) and scaled (scale ratio from 0.8 to 1.2) images; Dataset (ii) horizontal flip 50% images randomly; Dataset (iii) images with random contrast and brightness; Dataset (iv) the combination of (i), (ii) and (iii). In box plots, a box indicates the median and first and third quartile, whiskers indicate range of data and points indicate outliers. (b) Plots of Tukey's test (95% family-wise confidence level) on whether metric (IOU, precision and recall) differences among tested datasets (*n*=5094) are significantly different (blue: significance; grey: no significance) from 0 (red dotted lines). In Tukey test plots, a point indicates the mean and whiskers indicate the 95% confidence intervals. Source data are provided as a Source Data file.

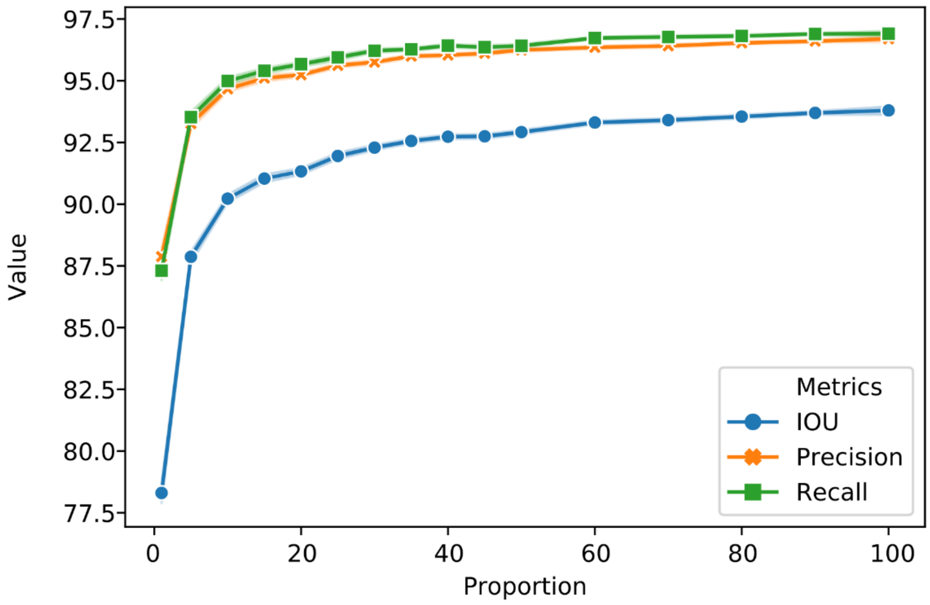

Supplementary Fig. 6. The performances (IOU, precision and recall) of the same validation set (*n*=1018) using 15 proportions (1%, every 5% from 5% to 50% and every 10% from 50% to 90%) of the original training set. Source data are provided as a Source Data file.

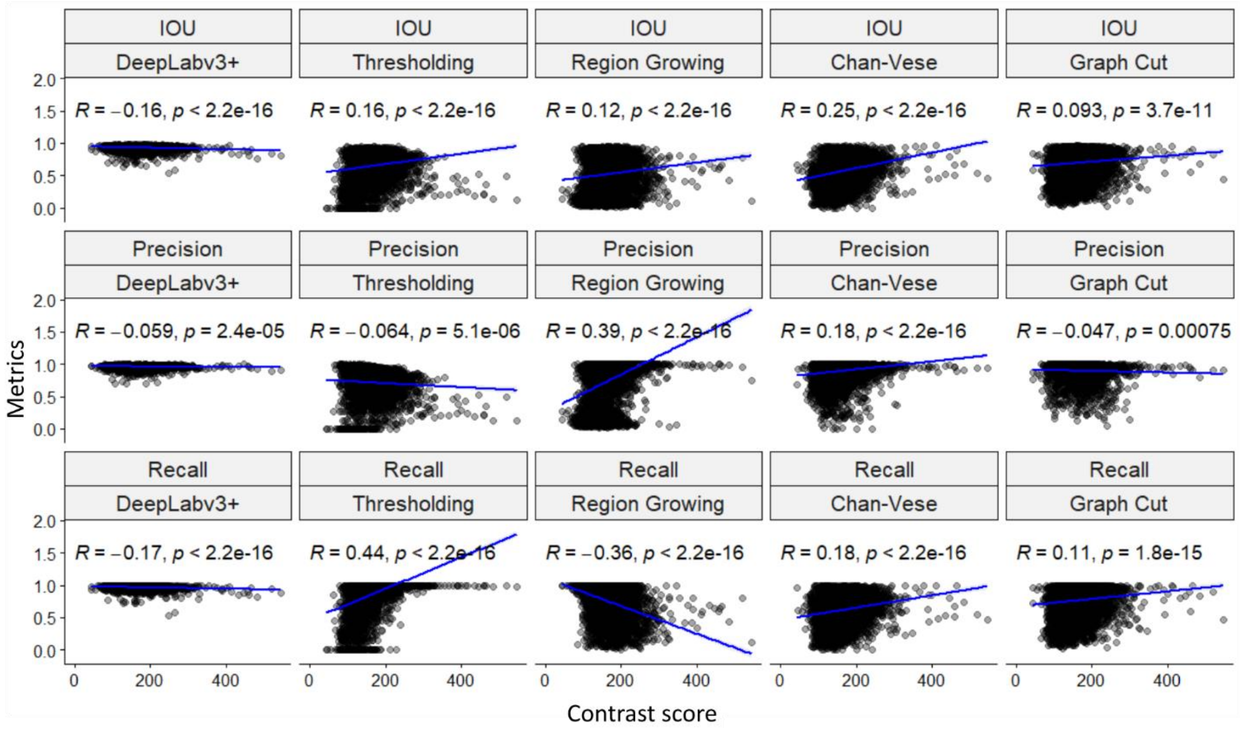

Supplementary Fig. 7. Performance metrics (from top to bottom: IOU, precision and recall) of predictions (*n*=5094) from different methods (from left to right: DeepLabv3+, thresholding, region growing, Chan-Vese, Graph Cut) in relation to the degree of contrast between plumage and non-plumage areas. Pearson correlation was used to test the correlation between performance and contrast. In all cases, tests were two-sided with no adjustments for multiple tests. Source data are provided as a Source Data file.

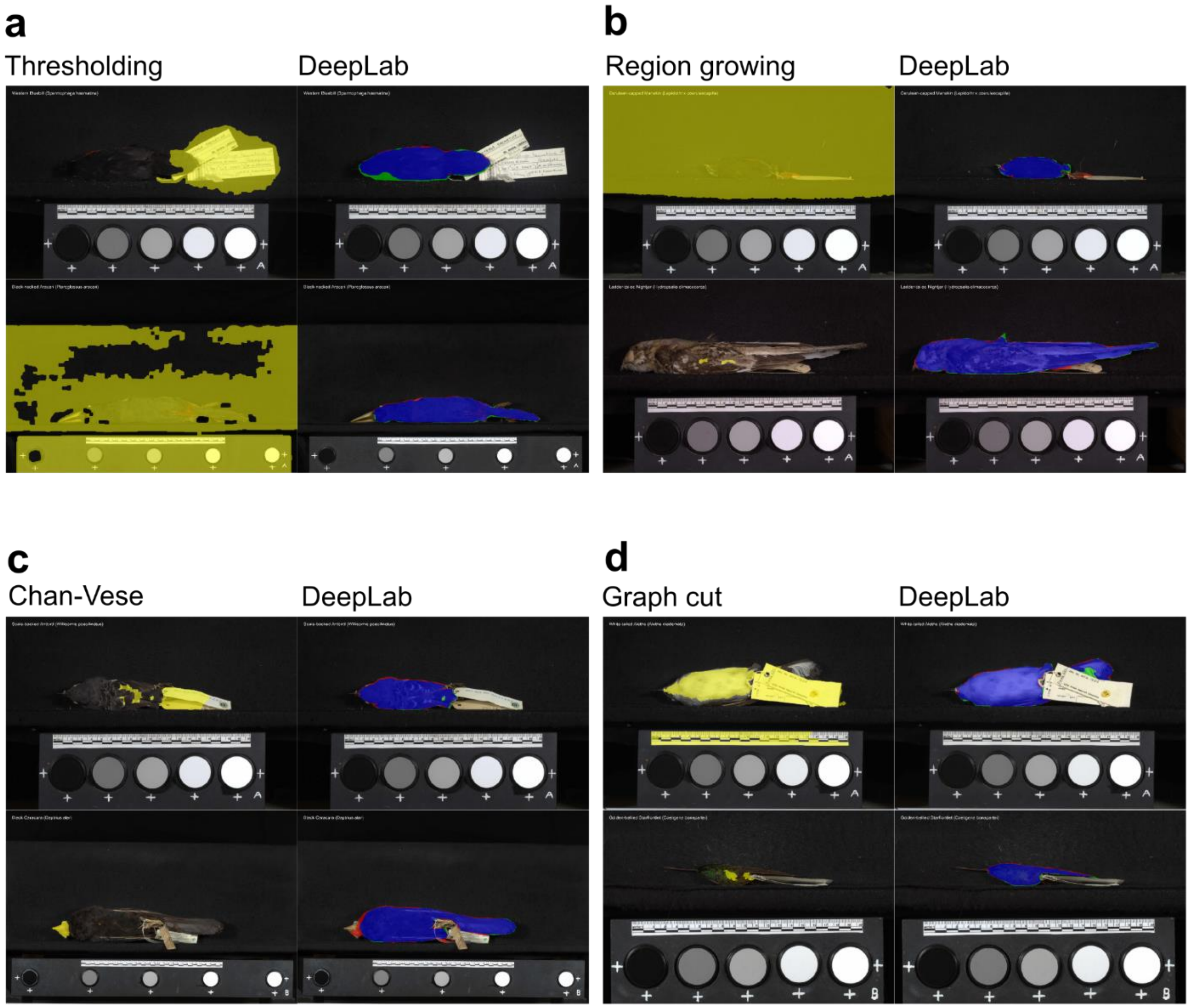

Supplementary Fig. 8. Examples of poorly segmented results from classic methods as well as deep learning predictions of the corresponding images. (a) Thresholding; (b) Region growing; (c) Chan-Vese; (d) Graph cut. Yellow is the segmentation from classic methods. Deep learning results are represented in blue, red and green as defined in Fig. 2.

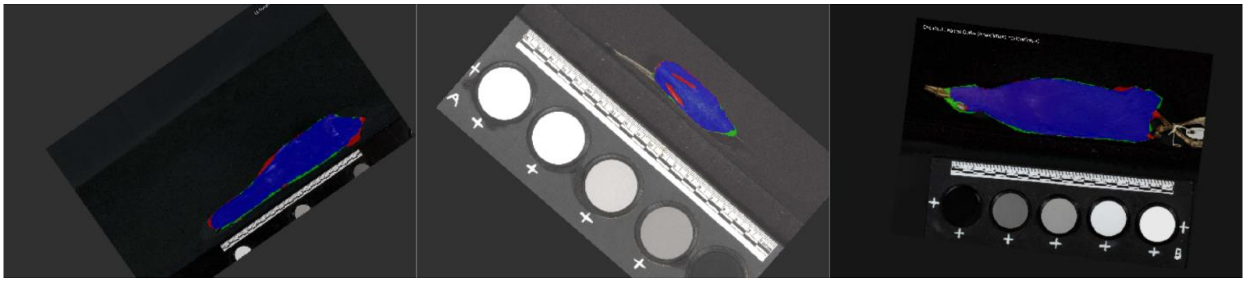

Supplementary Fig. 9. Examples of predictions using Dataset (iv) of the low-quality datasets. Images from Dataset (iv) were randomly rotated, translated, scaled, horizontal flipped and adjusted contrast and brightness.

Supplementary Tables

Supplementary Table 1. Overview of the four classic segmentation methods (thresholding, region growing, Chan-Vese, and graph cut) we tested on the avian specimen dataset.

Method	Description
Thresholding[13, 14]	Thresholding segments an image by allocating each pixel to either the foreground or the background by comparing the pixel value (e.g. grayscale value) to a pre-defined threshold value. The threshold can be set either manually or automatically calculated based on image features such as the image histogram or entropy.
Region growing[15]	Region growing first segments pixels that are the neighbour of the initial seeds (i.e. starting locations) into the foreground if the pixel value difference between an initial seed and a neighbour pixel is less than a pre-defined specific range. The algorithm keeps iterating this step until no pixel can be segmented.
Chan-Vese[16]	Chan-Vese requires a closed contour to be first placed (often manually) on the image. The algorithm then transforms the contour to minimise the sum of the internal (i.e. regions inside the contour) and external (i.e. regions outside the contour) energy (related to pixel values). After the energy is minimised, the region inside the final contour is the segmentation.
Graph cut[17]	Graph cut requires initial inputs of foreground and background seeds (pre-defined pixels that are from foreground or background). The algorithm treats an image as a graph, with pixels as the graph nodes. Every node (pixel) has three types of edges: (i) edges to the node's neighbour nodes; (ii) an edge to a source node (foreground); (iii) an edge to a sink node (background). Edge weights are based on pixel values and statuses (i.e. foreground, background or to be segmented). The algorithm segments an image by dividing the graph into two subgraphs (foreground and background) that have the largest sum of weights, and the segmentation is the foreground subgraph.

Supplementary Table 2. IOU, precision and recall of predictions from the DeepLabV3+ model. $n_{overall}$ = 5094, $n_{back/side/belly}$ = 1698.

Dataset	Mean	SD	Min	Max
IOU				
Overall	93.1	3.2	53.6	98.5
Back	94.6	2.8	67.6	98.5
Belly	91.9	3.4	53.6	97.2
Side	92.9	2.9	58.3	97.2
Precision				
Overall	96.3	2.4	70.1	99.9
Back	96.8	2.5	70.2	99.9
Belly	95.7	2.5	70.1	99.8
Side	96.4	2.0	70.9	99.9
Recall				
Overall	96.6	2.5	53.6	99.9
Back	97.6	2.0	72.0	99.9
Belly	95.8	2.7	53.6	99.6
Side	96.2	2.4	58.3	99.4

Supplementary Table 3. Phylogenetic heritability (H^2) estimates for UV colouration metrics across passerine birds (*n* = 4545). All models were run over 100 posterior phylogenetic trees.

Variable	Body region	Sex	H^2 (95% CI)
Average *u*	Dorsal	M	0.900 (0.876, 0.919)
		F	0.865 (0.836, 0.891)
	Ventral	M	0.852 (0.814, 0.880)
		F	0.809 (0.769, 0.848)
Peak *u* (Q50-100)	Dorsal	M	0.907 (0.881, 0.925)
		F	0.863 (0.832, 0.892)
	Ventral	M	0.871 (0.840, 0.902)
		F	0.828 (0.788, 0.863)
Peak *u* (Q75-100)	Dorsal	M	0.909 (0.883, 0.927)
		F	0.858 (0.827, 0.890)
	Ventral	M	0.882 (0.854, 0.914)
		F	0.834 (0.796, 0.868)
Peak *u* (Q90-100)	Dorsal	M	0.904 (0.877, 0.923)
		F	0.853 (0.815, 0.883)
	Ventral	M	0.890 (0.858, 0.918)
		F	0.826 (0.791, 0.865)
UV+ colouration (>1%)	Dorsal	M	0.905 (0.869, 0.943)
		F	0.899 (0.847, 0.927)
	Ventral	M	0.899 (0.855, 0.930)
		F	0.904 (0.859, 0.939)
UV+ colouration (>5%)	Dorsal	M	0.912 (0.854, 0.942)
		F	0.898 (0.830, 0.938)
	Ventral	M	0.927 (0.880, 0.958)
		F	0.914 (0.859, 0.947)
UV+ colouration (>10%)	Dorsal	M	0.920 (0.844, 0.946)
		F	0.896 (0.796, 0.940)
	Ventral	M	0.918 (0.865, 0.953)
		F	0.894 (0.819, 0.930)

Supplementary Table 4. Bayesian phylogenetic mixed model results for the effect of predictor variables on plumage UV reflectance in passerine species (n = 4527). All variables were standardised (mean = 0, sd = 1) prior to model fitting. M, male; UVS, ultraviolet sensitive. *, P < 0.05; **, P < 0.01; ***, P < 0.001. All models were run over 100 posterior phylogenetic trees. In all cases, tests were two-sided with no adjustments for multiple tests.

Variable	Term	Estimate (95% CI)	P_{MCMC}	$R^2_{marginal}$	$R^2_{conditional}$
Average *u*	(Intercept)	-0.076 (-1.033, 0.867)	0.876	0.06 (0.04, 0.10)	0.85 (0.83, 0.87)
	**Sex**$_{[M]}$	0.141 (0.133, 0.149)	<0.001		
	**Body region**$_{[Ventral]}$	-0.259 (-0.267, -0.251)	<0.001		
	**UVB radiation**	0.049 (0.023, 0.076)	<0.001		
	**Solar radiation**	-0.061 (-0.086, -0.036)	<0.001		
	**Temperature**	0.047 (0.015, 0.078)	0.003		
	**Forest dependency**$_{[High]}$	0.076 (0.053, 0.098)	<0.001		
	**Foraging strata**	0.041 (0.017, 0.065)	0.001		
	Visual system$_{[UVS]}$	0.144 (-0.128, 0.409)	0.292		
Peak *u* (Q50-100)	(Intercept)	-0.007 (-0.934, 0.952)	0.983	0.06 (0.04, 0.11)	0.86 (0.84, 0.87)
	**Sex**$_{[M]}$	0.168 (0.160, 0.176)	<0.001		
	**Body region**$_{[Ventral]}$	-0.200 (-0.208, -0.192)	<0.001		
	**UVB radiation**	0.066 (0.040, 0.092)	<0.001		
	**Solar radiation**	-0.069 (-0.093, -0.044)	<0.001		
	**Temperature**	0.050 (0.019, 0.081)	0.002		
	**Forest dependency**$_{[High]}$	0.090 (0.067, 0.112)	<0.001		
	**Foraging strata**	0.038 (0.015, 0.063)	0.002		
	Visual system$_{[UVS]}$	0.188 (-0.099, 0.473)	0.198		
Peak *u* (Q75-100)	(Intercept)	0.067 (-0.880, 1.032)	0.891	0.07 (0.03, 0.12)	0.87 (0.85, 0.88)
	**Sex**$_{[M]}$	0.175 (0.168, 0.183)	<0.001		
	**Body region**$_{[Ventral]}$	-0.159 (-0.166, -0.151)	<0.001		
	**UVB radiation**	0.071 (0.045, 0.097)	<0.001		
	**Solar radiation**	-0.070 (-0.094, -0.045)	<0.001		
	**Temperature**	0.050 (0.018, 0.081)	0.002		
	**Forest dependency**$_{[High]}$	0.096 (0.073, 0.118)	<0.001		
	**Foraging strata**	0.033 (0.009, 0.057)	0.006		
	Visual system$_{[UVS]}$	0.222 (-0.115, 0.535)	0.182		
Peak *u* (Q90-100)	(Intercept)	0.163 (-0.802, 1.110)	0.736	0.07 (0.03, 0.13)	0.87 (0.85, 0.88)
	**Sex**$_{[M]}$	0.172 (0.165, 0.180)	<0.001		
	**Body region**$_{[Ventral]}$	-0.126 (-0.134, -0.118)	<0.001		
	**UVB radiation**	0.072 (0.046, 0.098)	<0.001		
	**Solar radiation**	-0.069 (-0.094, -0.045)	<0.001		
	**Temperature**	0.054 (0.023, 0.086)	0.001		
	**Forest dependency**$_{[High]}$	0.098 (0.075, 0.121)	<0.001		
	**Foraging strata**	0.027 (0.003, 0.051)	0.028		
	Visual system$_{[UVS]}$	0.247 (-0.111, 0.596)	0.170		
UV+ colouration (>1%)	(Intercept)	-4.202 (-9.426, 1.136)	0.115	0.03 (0.01, 0.08)	0.92 (0.90, 0.93)
	**Sex**$_{[M]}$	0.469 (0.411, 0.527)	<0.001		
	**Body region**$_{[Ventral]}$	0.213 (0.156, 0.271)	<0.001		
	UVB radiation	0.072 (-0.118, 0.255)	0.449		
	**Solar radiation**	-0.255 (-0.412, -0.096)	0.002		
	**Temperature**	0.443 (0.222, 0.658)	<0.001		
	**Forest dependency**$_{[High]}$	0.217 (0.071, 0.360)	0.003		
	**Foraging strata**	0.393 (0.239, 0.543)	<0.001		
	Visual system$_{[UVS]}$	0.874 (-0.526, 2.325)	0.224		
UV+ colouration (>5%)	(Intercept)	-6.705 (-12.521, -0.893)	0.022	0.04 (0.01, 0.08)	0.93 (0.92, 0.94)
	**Sex**$_{[M]}$	0.460 (0.392, 0.532)	<0.001		
	**Body region**$_{[Ventral]}$	0.434 (0.363, 0.503)	<0.001		
	UVB radiation	0.013 (-0.220, 0.240)	0.914		
	**Solar radiation**	-0.330 (-0.521, -0.134)	0.001		
	**Temperature**	0.587 (0.312, 0.859)	<0.001		
	**Forest dependency**$_{[High]}$	0.263 (0.088, 0.436)	0.003		
	**Foraging strata**	0.458 (0.274, 0.642)	<0.001		
	Visual system$_{[UVS]}$	0.950 (-0.537, 2.420)	0.203		
UV+ colouration (>10%)	(Intercept)	-7.647 (-13.316, -1.983)	0.007	0.04 (0.02, 0.08)	0.92 (0.91, 0.94)
	**Sex**$_{[M]}$	0.475 (0.398, 0.553)	<0.001		
	**Body region**$_{[Ventral]}$	0.634 (0.556, 0.714)	<0.001		
	UVB radiation	0.091 (-0.144, 0.331)	0.455		
	**Solar radiation**	-0.350 (-0.554, -0.145)	0.001		
	**Temperature**	0.574 (0.289, 0.865)	<0.001		
	**Forest dependency**$_{[High]}$	0.225 (0.039, 0.408)	0.017		
	**Foraging strata**	0.489 (0.294, 0.682)	<0.001		
	Visual system$_{[UVS]}$	0.899 (-0.507, 2.309)	0.207		

Supplementary references

1. Chen LC, Papandreou G, Kokkinos I, Murphy K, Yuille AL. DeepLab: semantic image segmentation with deep convolutional nets, atrous convolution, and fully connected CRFs. *arXiv* **1606**, 00915 (2017).

2. Kim J, Lee JK, Lee KM. Accurate image super-resolution using very deep convolutional networks. *arXiv* **1511**, 04587 (2015).

3. Basu S, Ganguly S, Mukhopadhyay S, DiBiano R, Karki M, Nemani R. DeepSat - a learning framework for satellite imagery. In: *Proceedings of the 23rd SIGSPATIAL International Conference on Advances in Geographic Information Systems*) (2015).

4. Potena C, Nardi D, Pretto A. Fast and accurate crop and weed identification with summarized train sets for precision agriculture. In: *Intelligent Autonomous Systems 14. IAS 2016. Advances in Intelligent Systems and Computing, vol 531.* (eds Chen W, Hosoda K, Menegatti E, Shimizu M, Wang H). Springer, Cham (2017).

5. Milioto A, Lottes P, Stachniss C. Real-time semantic segmentation of crop and weed for precision agriculture robots leveraging background knowledge in CNNs. In: *2018 IEEE International Conference on Robotics and Automation*) (2018).

6. Hudson LN*, et al.* Inselect: automating the digitization of natural history collections. *PloS one* **10**, e0143402 (2015).

7. Bradski G. The OpenCV Library. *Dr Dobb's Journal of Software Tools* **120**, 122-125 (2000).

8. Joulin A, van der Maaten L, Jabri A, Vasilache N. Learning visual features from large weakly supervised data. *arXiv* **1511**, 02251 (2015).

9. Hestness J*, et al.* Deep learning scaling is predictable, empirically. *arXiv* **1712**, 00409 (2017).

10. Berzins V. Accuracy of laplacian edge detectors. *Computer Vision, Graphics, and Image Processing* **27**, 195-210 (1984).

11. van Vliet LJ, Young IT, Beckers GL. A nonlinear laplace operator as edge detector in noisy images. *Computer Vision, Graphics, and Image Processing* **45**, 167-195 (1989).

12. Haralick RM, Sternberg SR, Zhuang X. Image analysis using mathematical morphology. *IEEE Transactions on Pattern Analysis and Machine Intelligence* **9**, 532-550 (1987).

13. Kohler R. A segmentation system based on thresholding. *Computer Graphics and Image Processing* **15**, 319-338 (1981).

14. Otsu N. A threshold selection method from gray-level histograms. *IEEE Transactions on Systems, Man, and Cybernetics* **9**, 62-66 (1979).

15. Adams R, Bischof L. Seeded region growing. *IEEE Transactions on Pattern Analysis and Machine Intelligence* **18**, 641-647 (1994).

16. Chan TF, Vese LA. Active contours without edges. *IEEE Transactions on Image Processing* **10**, 266-277 (2001).

17. Boykov YY, Jolly MP. Interactive graph cuts for optimal boundary & region segmentation of objects in N-D images. In: *Proceedings Eighth IEEE International Conference on Computer Vision*) (2001).
