# Supplementary material for: Deep learning image segmentation reveals patterns of UV reflectance evolution in passerine birds
Source: Nat Commun. 2022 Aug 29;13:5068. doi: 10.1038/s41467-022-32586-5 (PMC9424304; doi:10.1038/s41467-022-32586-5)
Supplement: Supplementary file 3 — Reporting Summary [file 41467_2022_32586_MOESM3_ESM.pdf]

Corresponding author(s): Yichen He

Last updated by author(s): Jul 27, 2022

Reporting Summary

Nature Portfolio wishes to improve the reproducibility of the work that we publish. This form provides structure for consistency and transparency in reporting. For further information on Nature Portfolio policies, see our Editorial Policies and the Editorial Policy Checklist.

Statistics

For all statistical analyses, confirm that the following items are present in the figure legend, table legend, main text, or Methods section.

n/a	Confirmed	
☐	☒	The exact sample size (*n*) for each experimental group/condition, given as a discrete number and unit of measurement
☐	☒	A statement on whether measurements were taken from distinct samples or whether the same sample was measured repeatedly
☐	☒	The statistical test(s) used AND whether they are one- or two-sided<br>*Only common tests should be described solely by name; describe more complex techniques in the Methods section.*
☐	☒	A description of all covariates tested
☐	☒	A description of any assumptions or corrections, such as tests of normality and adjustment for multiple comparisons
☐	☒	A full description of the statistical parameters including central tendency (e.g. means) or other basic estimates (e.g. regression coefficient) AND variation (e.g. standard deviation) or associated estimates of uncertainty (e.g. confidence intervals)
☐	☒	For null hypothesis testing, the test statistic (e.g. F, t, r) with confidence intervals, effect sizes, degrees of freedom and P value noted<br>*Give P values as exact values whenever suitable.*
☐	☒	For Bayesian analysis, information on the choice of priors and Markov chain Monte Carlo settings
☐	☒	For hierarchical and complex designs, identification of the appropriate level for tests and full reporting of outcomes
☐	☒	Estimates of effect sizes (e.g. Cohen's d, Pearson's r), indicating how they were calculated

Our web collection on statistics for biologists contains articles on many of the points above.

Software and code

Policy information about availability of computer code

Data collection	No custom software used for data collection.
Data analysis	Data were analysed in R (version 4.1.0) using the open-source packages cited in the text (raster 3.4-5, pavo 2.6.1, phangorn 2.5.5, MCMCglmm 2.32). We also used DCRAW (version 9.27) to linearise the digital image data and the ImageJ Multispectral Image Calibration and Analysis Toolbox (version 2.2) to generate cone catch mapping functions. The ground truth (expert-labelled) segmentations were labelled using Zooniverse (http://www.projectplumage.org). Python (3.7.6) along with Tensorflow (1.6.0), scikit-image (0.16.2) and opencv-python (4.1.1.26) were used to conduct image processing, deep learning and other segmentation methods (thresholding, region growing, graph cut are from the opencv-python library. Chan-vese is from scikit-image library) in the study. There software and codes are open sources and available from the sources listed in the text. The analysis code in this study is available at https://github.com/EchanHe/DL_seg_avian_plumage.

For manuscripts utilizing custom algorithms or software that are central to the research but not yet described in published literature, software must be made available to editors and reviewers. We strongly encourage code deposition in a community repository (e.g. GitHub). See the Nature Portfolio guidelines for submitting code & software for further information.

Data

Policy information about availability of data

All manuscripts must include a data availability statement. This statement should provide the following information, where applicable:

- Accession codes, unique identifiers, or web links for publicly available datasets
- A description of any restrictions on data availability
- For clinical datasets or third party data, please ensure that the statement adheres to our policy

The dataset used in this paper is available at https://doi.org/10.15131/shef.data.19221699, including specimen images, expert labelled images and machine-predicted segmentations associated with training and testing our deep learning model. Avian phylogenetic trees were downloaded from http://www.birdtree.org, species' geographical range maps and ecological data were accessed via BirdLife International's Data Zone (http://www.datazone.birdlife.org). Global climate data were downloaded from WorldClim (https://worldclim.org/)

Field-specific reporting

Please select the one below that is the best fit for your research. If you are not sure, read the appropriate sections before making your selection.

☐ Life sciences ☐ Behavioural & social sciences ☒ Ecological, evolutionary & environmental sciences

For a reference copy of the document with all sections, see nature.com/documents/nr-reporting-summary-flat.pdf

Ecological, evolutionary & environmental sciences study design

All studies must disclose on these points even when the disclosure is negative.

Study description	The manuscript uses deep learning to collect bird plumage UV and addresses UV in passerine birds.
Research sample	Our research sample consisted of individual specimens (males and females) of 4,527 passerine bird species. Sample selection was determined by the availability of museum specimens. We used all the available passerine specimens for this research. The sample of 4,527 passerine bird species represents more than 65% of the total passerine bird species (~6,500 species).
Sampling strategy	We collected plumage colouration data for up to three males and three female of all passerine bird species housed at the NHM, Tring. Final sample size was determined by specimen availability.
Data collection	Plumage UV was measured using calibrated digital images of individual specimens. Data for predictor variables were sourced from the literature. Digital images were taken by CRC, ZKV, LON, CJAM and MDJ. Segmentations were labelled by YH and CRC.
Timing and spatial scale	Plumage UV data were sampled from the collections at NHM Tring during the period 2016-2019. The dataset is global in spatial scale.
Data exclusions	No data were excluded from the analyses.
Reproducibility	Ours is a non-experimental study.
Randomization	Randomisation was not used in sample selection. Sample selection was determined by the availability of museum specimens. For training the deep learning model, expert labelled images were randomly sampled into the training set and the validation set.
Blinding	Blinding was not relevant to this study as species sampling was as exhaustive as possible and species were sampled without regard to their phenotypes.

Did the study involve field work? ☐ Yes ☒ No

Reporting for specific materials, systems and methods

We require information from authors about some types of materials, experimental systems and methods used in many studies. Here, indicate whether each material, system or method listed is relevant to your study. If you are not sure if a list item applies to your research, read the appropriate section before selecting a response.

Materials & experimental systems

n/a	Involved in the study
☒	☐ Antibodies
☒	☐ Eukaryotic cell lines
☒	☐ Palaeontology and archaeology
☒	☐ Animals and other organisms
☒	☐ Human research participants
☒	☐ Clinical data
☒	☐ Dual use research of concern

Methods

n/a	Involved in the study
☒	☐ ChIP-seq
☒	☐ Flow cytometry
☒	☐ MRI-based neuroimaging
